# Effect of acute exercise on the dynamics of testosterone levels: a systematic review of randomized controlled trials

**DOI:** 10.7717/peerj.20615

**Published:** 2026-01-08

**Authors:** Qianxin Tu, Gen Li, Songtao Wang

**Affiliations:** School of Physical Education and Sports Science, South China Normal University, Guangzhou, Panyu, China

**Keywords:** Acute exercise, Testosterone, Recovery period

## Abstract

**Purpose:**

Although acute exercise has been demonstrated to modulate endogenous testosterone levels, existing studies have reached conflicting conclusions regarding the pattern of response of testosterone levels after exercise. The objective of this study was to examine the dynamic effects of acute exercise on testosterone levels and to analyze the differences in the role of factors such as exercise mode, intensity, sample source, and gender.

**Methods:**

A comprehensive search of articles published up to March 2025 was conducted in five database systems, including PubMed and Web of Science, in accordance with the PRISMA guidelines. A total of 15 randomized controlled trials assessing the effect of acute exercise on testosterone levels were included, with a total sample size of 251 participants.

**Results:**

(1) Testosterone levels demonstrate a greater increase following resistance training, with a delayed return to baseline levels; in contrast, testosterone levels typically recover within 1 h after aerobic exercise. (2) Moderate to high-intensity exercise stimulates the hypothalamic-pituitary-gonadal axis (HPG), leading to a transient rise in testosterone, but extended high-intensity exercise causes testosterone suppression during recovery due to cortisol antagonism for up to 72 h. The testosterone concentration during the active phase exceeds that during the recovery period. (3) A significant disparity in baseline testosterone levels exists between males and females, with males exhibiting higher levels. Additionally, males demonstrate a more pronounced response to exercise compared to females. (4) Blood tests exhibit greater sensitivity than saliva tests, although the latter is more reactive to high-intensity exercise; (5) The response is more pronounced in younger males compared to older adults, with negligible response observed in adolescents.

**Conclusion:**

Variations in testosterone level modulation due to acute exercise are predominantly influenced by exercise mode, intensity, sample source, and subject characteristics (gender and age). While resistance training and high-intensity exercise might temporarily increase testosterone levels, it is essential to consider the potential for hormonal imbalance after recovery; age and sex variations, along with assay standardization, are critical areas for further investigation. This review was registered PROSPERO with registration number CRD420251007222.

## Introduction

Testosterone is a primary androgenic steroid hormone synthesized from cholesterol through a series of enzymatic reactions. In males, the primary site of secretion is the leydig cells within the testes. In females, testosterone is secreted at much lower levels by the ovaries and adrenal cortex. Besides its crucial involvement in reproductive function and fertility preservation, it has systemic regulatory effects on other physiological systems, including the maintenance of musculoskeletal homeostasis (Bhasin et al., 1996), cognitive control (McHenry et al., 2014), and cardiometabolic health (Tirabassi et al., 2013).

Testosterone is identified as a crucial factor in preserving muscle mass and function throughout the ageing process (Galvão et al., 2008). Nonetheless, subsequent to the third decade of life, testosterone levels progressively diminish at a rate of 1–2% year (Tajar et al., 2010), a phenomenon primarily ascribed to age-related hypogonadism. This endocrine modification is clinically significant, as epidemiological investigations have established an independent correlation between diminished testosterone levels and the dysregulation of metabolic and inflammatory pathways (Tang et al., 2007).

Testosterone deficiency syndrome (TDS) clinically manifests with several detrimental effects, including as erectile dysfunction, reduced sperm motility, and heightened cardiovascular morbidity and overall mortality (Halpern & Brannigan, 2019; Yeap et al., 2024). While exogenous testosterone injection is the principal therapeutic approach for TDS, its non-medical misuse, particularly in competitive athletics, has generated considerable public health issues. The usage of anabolic androgenic steroids (AAS), including illegal testosterone analogues, is linked to significant cardiometabolic consequences, neuropsychiatric problems, and elevated mortality rates (Horwitz, Andersen & Dalhoff, 2019; Smoliga, Wilber & Robinson, 2023). Consequently, regulatory bodies like the World Anti-Doping Agency (WADA) categorically forbid the utilization of testosterone and its derivatives to uphold the integrity of athletes and ensure participant safety.

Testosterone levels exhibit a dynamic response to lifestyle modifications, with physical exercise emerging as a significant moderator. Epidemiological evidence indicates that age-related reductions in testosterone are inversely correlated with levels of regular physical activity (Del Giudice et al., 2021). Mechanistic studies indicate that both resistance training and aerobic exercise may mitigate hypogonadal tendencies *via* exercise-induced biochemical changes (Kumagai et al., 2018; Räntilä, Ahtiainen & Häkkinen, 2023). The anabolic effects of these exercise modalities are hypothesized to include complex endocrine interactions, potentially mediated by acute hormonal fluctuations and persistent metabolic reprogramming.

Recent research highlights the intricate link between acute exercise sessions and endocrine regulation, specifically with testosterone dynamics (Chatzinikolaou et al., 2010; Luk et al., 2019; McCaulley et al., 2009). Proposed mechanisms indicate that exercise-induced stress reactions modulate testosterone secretion *via* the activation of the hypothalamic-pituitary-gonadal(HPG) axis (Foresta et al., 1997), which is considered a primary regulator of testosterone anabolic activities (Le et al., 2014). The increase in testosterone after resistance or aerobic exercise has been extensively recorded (Kraemer & Ratamess, 2005), with possible factors including (1) temporary elevation resulting from hemoconcentration due to decreased plasma volume (Jezová & Vigas, 1981), and (2) lactate-induced stimulation of steroidogenesis acute regulatory protein expression (Lin et al., 2001). Sympathoadrenal stimulation enhances the responsiveness of testicular mesenchymal cells (Fry & Kraemer, 1997).

Scholars are increasingly concentrating on the dynamic variations of testosterone during post-exercise recovery. Despite the circadian regularity of testosterone secretion, which peaks between 05:30 and 08:00 and thereafter declines to a minimum over 12 h (Brambilla et al., 2009), acute exercise seems to influence this temporal pattern. A temporary increase in testosterone is typically noted immediately following the cessation of exercise, with peak levels generally aligning with the end of the activity (Zitzmann & Nieschlag, 2001). Nonetheless, findings have been variable, with certain studies indicating sustained testosterone levels below baseline for hours or even days post-exercise (Daly et al., 2005), underscoring the intricacy of hormone recovery kinetics. Systematic assessments since 2014 have underscored this inconsistency. For instance, O’Leary & Hackney (2014) documented a persistent elevation in testosterone levels following resistance training, but Hayes et al. (2015) saw no significant alteration in testosterone levels post-exercise. D’Andrea et al. (2020) posited that low-intensity exercise does not affect testosterone concentrations; nevertheless, extended low-intensity exercise may lead to a substantial increase in testosterone levels (Galbo et al., 1977). The observed heterogeneity may arise from various confounding factors, including exercise variability (differences in intensity, mode, and duration), individual susceptibility factors (age, gender, body composition, exercise experience), and discrepancies in sample sources (saliva, blood). Furthermore, the researchers failed to consider inter-study variations in baseline testosterone levels among subjects.

Investigating the fluctuations in blood testosterone levels post-exercise holds substantial physiological and practical importance. In training monitoring, blood testosterone serves as a crucial indicator of the body’s anabolic potential, with its fluctuations illustrating the body’s response to exercise load. Consequently, it is frequently utilized to evaluate training intensity and recovery from fatigue. Significantly, as a crucial androgen and anabolic hormone, blood testosterone is integral to the regulation of numerous physiological functions and metabolic processes, including lipolysis, body fat distribution, muscle protein synthesis, bone metabolism, erythropoiesis, and immune regulation. A comprehensive examination of the fluctuations in blood testosterone following acute exercise elucidates the physiological adaptation mechanisms induced by exercise and offers a foundational theoretical framework for understanding fatigue, physical recovery, and alterations in health status from a dynamic perspective. This insight is crucial for the formulation of personalized exercise regimens and evidence-based nutritional intervention strategies.

In light of these findings, to address the current contradictions in researchs and to investigate the dynamic effects of acute exercise on testosterone levels,the present study employed a rigorous randomized controlled trial (RCT) systematic evaluation of ∆T (change from baseline after exercise) as a standardized metric. Subsequent subgroup analyses were conducted to delineate suitable exercise regimens for modulating testosterone levels in specific populations.

## Methods

### Design

This systematic review adhered to the Cochrane Handbook of Selection and the Preferred Reporting Items for Systematic Reviews and Meta-Analyses (PRISMA) criteria (Page et al., 2021). The protocol is registered with PROSPERO under the registration number CRD420251007222.

### Search strategy

A literature search was performed in five principal databases: PubMed, Web of Science, Cochrane, Scopus, and Ebsco, as of March 31, 2025. The search procedure utilized keywords and Medical Subject Headings (MESH): ((Exercises OR Post-Exercise OR Acute Exercise OR After a Game) AND (Testosterone) AND (Randomized Controlled Trial)). Furthermore, to improve the search outcomes, the reference lists of chosen research were manually examined to discover potentially qualifying articles. Two authors (G.L. and Q.T.) independently searched for and screened studies according to predetermined criteria during the month of April, and duplicate articles were removed using EndNote 20. In the event of a disagreement, a third author (S.W.) was consulted until a consensus was achieved.

### Inclusion and exclusion criteria

The inclusion criteria comprised (1) a RCT design, (2) the presence of both an intervention and a control group, and (3) the measurement of testosterone as an end variable, (3) indexed in the Journal Citation Reports (JCR).

The exclusion criteria were: (1) review articles and conference proceedings; (2) research utilizing animal models; (3) publications with a significant risk of bias or without full-text availability; and (4) outcome measures that could not be transformed into mean and standard deviation.

The studies reviewed included only healthy individuals, and the research encompassed all age groups, males, and females.

### Data extraction

Two separate researchers (Q.T. and L.G.) employed a standardized technique in Microsoft Excel 2019 (Microsoft Corp., Redmond, WA, USA) for data extraction (refer to Table 1). The subsequent fields were methodically recorded: (1) Study characteristics: first author, year of publication, and sample size; (2) Intervention information: kind, duration, and intensity; and (3) Participant characteristics: age, gender, exercise experience, and percentage of body fat (%). All numerical data were verified against the original sources to ensure precision. Any differences that emerged during this procedure were addressed through consultation with a third researcher (S.W.).

**Table 1:** Characteristics of the studies included in this review.

Author and published year	Country	Sample size	Participants	Gender (Male/Female)	Age (Baseline)	Body fat (%) (Baseline)	Exercise intervention information			Sample	Time after exercise completion sample taken
							Exercise type	Intensity	Exercise duration		
Bekris et al. (2022)	Greece	INT: 12	Trained	M: 22	INT: 22.42 ± 3.96	INT: 15.31 ± 5.01	Aerobic exercise: small-sided game	High	45	Serum	Immediately, 24 h, 48 h, 72 h
		CON: 10		F: 0	CON: 22.20 ± 4.02	CON: 13.04 ± 3.53					
Chatzinikolaou et al. (2010)	Greece	INT: 12	Recreationally active	M: 24	INT: 23.1 ± 2.6	13.3 ± 1.6	Resistance exercise: plyometric exercises, 50 jumps over 50-cm hurdles (5 sets of 10 repetitions) and 50 drop jumps from 50-cm plyometric box (five sets of 10 repetitions)	Moderate	40	Serum	Immediately, 24 h, 48 h, 72 h, 96 h, 120 h
		CON: 12		F: 0	CON: 25.5 ± 1.9						
Copeland, Consitt & Tremblay (2002)-a	Canada	INT: 30	Recreationally active	M: 0	19–69	24.5 ± 5.2–	Aerobic exercise: 40 min of cycling at 75% maximum heart rate	High	40	Serum	Immediately, 30 min
		CON: 30		F: 30		29.6 ± 5.5					
Copeland, Consitt & Tremblay (2002)-b	Canada	INT: 30	Recreationally active	M: 0	19–69	24.5 ± 5.2–29.6 ± 5.5	Resistance exercise: three sets of 10 repetitions of eight exercises	Moderate	30-40	Serum	Immediately, 30min
		CON: 30		F: 30							
Hackney, Premo & McMurray (1995)-a	America	INT: 9	Trained	M: 9	30.6 ± 3.8	7.1 ± 2.4	Aerobic exercise: 1 h continuous aerobic exercise (65% VO_2_ max)	High	60	Serum	Immediately, 1 h, 2 h, 3 h, 4 h, 5 h, 6 h, 7 h, 8 h
		CON: 9		F: 0							
Hackney, Premo & McMurray (1995)-b	America	INT: 9	Trained	M: 9	30.6 ± 3.8	7.1 ± 2.4	Aerobic exercise: 1 h intermittent anaerobic exercise (which included 2 min exercise periods at 110% VO_2_ max)	High	60	Serum	Immediately, 1 h, 2 h, 3 h, 4 h, 5 h, 6 h, 7 h, 8 h
		CON: 9		F: 0							
Hough et al. (2011)-a	England	INT: 10	Trained	M: 10	24 ± 3	NA	Aerobic exercise: (a) continuous cycle tofatigue at 75% peak poweroutput; (b) 30-min cyclealternating 1-min 60% and 1 min 90% peakpower output; (c) 30-min cycle alternating 1-min 55% and 4-min 80% peak power output	High	30	Plasma and Saliva	Immediately, 10 min, 20 min, 30 min, 40 min, 50 min, 60 min
		CON: 30		F: 30							
Hough et al. (2011)-b	England	INT: 10	Trained	M: 10	24 ± 3	NA	Resistance exercise: Squatting eight sets of 10 repetitions at 10 repetition maximum	High	30	Plasma and Saliva	Immediately, 10 min, 20 min, 30 min, 40 min, 50 min, 60 min
		CON: 10		F: 0							
Ispirlidis et al. (2008)	Greece	INT: 14	Highly trained	M: 0	20.1 ± 0.8	NA	Aerobic exercise: soccer game	High	68	Serum	Immediately, 24 h, 48 h, 72 h, 96 h, 120 h, 144 h
		CON: 10		F: 24							
Knatauskaitė et al. (2022)-a	Germany	INT: 13 (M: 5 F: 8)	Recreationally active	M: 9	INT: 15.46 ± 0.52	NA	Aerobic exercise: game-based exercise	Moderate	20	Saliva	Immediately, 20 min, 60 min
		CON:12 (M: 4 F: 8)		F: 16	CON: 14.92 ± 0.29						
Knatauskaitė et al. (2022)-b	Germany	INT: 12 (M: 4 F: 8)	Recreationally active	M: 8	INT: 15.17 ± 0.39	NA	Aerobic exercise: game-based exercise	High	20	Saliva	Immediately, 20 min, 60 min
		CON: 12 (M: 4 F: 8)		F: 16	CON: 14.92 ± 0.29						
Kraemer et al. (2001)	America	INT: 5	Trained	M: 10	21.6 ± 1.1	7.9 ± 1.7	Resistance exercise: four sets of 10-repetition-maximum, hang pulls, bench press, leg press, seated row, leg curl, military press, lat pulls, knee extensions, arm curls, sit-ups	Moderate	90	Saliva	10 min, 70 min, 130 min, 190 min, 250 min, 310 min, 370 min, 430 min, 490 min, 550 min, 610 min, 670 min, 730 min, 790 min, 850 min
		CON: 5		F: 0							
Lane & Hackney (2015)-a	America	INT: 12	Trained	M: 12	22 ± 4.6	8.4 ± 2.1	Aerobic exercise: 40% VO_2_max cycling	Moderate	30	Serum and Saliva	Immediately, 30 min
		CON: 12		F: 0							
Lane & Hackney (2015)-b	America	INT: 12	Trained	M: 12	22 ± 4.6	8.4 ± 2.1	Aerobic exercise: 60% VO_2_max cycling	Moderate	30	Serum and Saliva	Immediately, 30 min
		CON: 12		F: 0							
Lane & Hackney (2015)-c	America	INT: 12	Trained	M: 12	22 ± 4.6	8.4 ± 2.1	Aerobic exercise: 80% VO_2_max cycling	High	45	Serum and Saliva	Immediately, 30 min
		CON: 12		F: 0							
McCaulley et al. (2009)-a	America	INT: 10	Trained	M: 10	21.8 ± 1.9	13.2 ± 4.2	Resistance exercise: four sets of 10 repetitions 75% 1-RM, squats (hypertrophy)	Moderate	15	Serum	Immediately, 60 min
		CON: 10		F: 0							
McCaulley et al. (2009)-b	America	INT: 10	Trained	M: 10	21.8 ± 1.9	13.2 ± 4.2	Resistance exercise: 11 sets of three repetitions 90% 1-RM, squats (strength)	High	70	Serum	Immediately, 60 min
		CON: 10		F: 0							
McCaulley et al. (2009)-c	America	INT: 10	Trained	M: 10	21.8 ± 1.9	13.2 ± 4.2	Resistance exercise: eight sets of six repetitions 0% 1-RM, jump squats (power)	High	30	Serum	Immediately, 60 min
		CON: 10		F: 0							
Mirazizi, Rahmati-Ahmadabad & Fatolahi (2022)	Iran	INT: 15	Highly trained	M: 30	19–22	NA	Aerobic exercise: agility ladder exercise	Moderate	45	Serum	Immediately, 24 h, 48 h
		CON: 15		F: 0							
Ronsen et al. (2001)	Norway	INT: 9	Highly trained	M: 9	21–27	NA	Aerobic exercise: 50% VO_2_max cycling	Moderate	65	Serum	Immediately, 15 min, 30 min, 60 min, 2 h, 3 h, 4 h
		CON: 9		F: 0							
Schumm et al. (2008)	America	INT: 9	Recreationally active	M: 9	26.2 ± 8.7	15.1 ± 5.35	Resistance exercise: four sets of 10-repetition-maximum, Smith machine squats	Moderate	10	Serum	Immediately, 15 min, 30, 60 min
		CON: 9		F: 0							
Shariat et al. (2014)	Malaysia	INT: 10	Highly trained	M: 10	18.0 ± 1.3	21.40 ± 3.65	Resistance exercise: three sets of 6–7 repetitions 85% 1-RM hang pulls, bench press, leg press, seated row, leg curl, shoulder press, lat pulls, knee extensions, arm curls, sit-ups	High	90	Saliva	Immediately, 30 min, 2.5, 4.5 h
		CON: 10		F: 0							
Willoughby & Taylor (2004)	America	INT: 9	Sedentary	M: 9	INT: 19.36 ± 2.17	NA	Resistance exercise: three sets of 8–10 repetitions 75–80% 1-RM squat, leg press, leg extension	Moderate	50	Serum	Immediately, 30 min
		CON: 9		F: 0	CON: 20.66 ± 1.82						
Xu & Guo (2025)	China	INT: 12	Recreationally active	M: 12	26.2 ± 4.4	NA	Resistance exercise: depth jump, 15 sets of 10 repetitions	Moderate	45	Serum	Immediately, 60 min
		CON: 12		F: 0							

**Note:**

INT, intervene; CON, control group; M, male; F, female; 1-RM, one-repetition maximum; VO_2_max, maximal oxygen consumption; NA, not available. The letters a, b, and c following the author and publication year denote different intervention methods employed within the same study.

### Quality assessment

Two researchers (G.L. and Q.T.) independently performed bias evaluations use the Cochrane Risk of Bias Tool for Randomized Controlled Trials (RoB-2) (Zhang et al., 2025). The evaluation encompassed six domains: randomization sequence creation, allocation concealment, blinding (of participants/staff and outcome assessors), measurement bias, inadequate outcome data, reporting bias, and additional potential biases. Rating differences were resolved through organized conversations with an external methodologist (S.W.). Each element was classified according to the official RoB-2 decision-making algorithm as “low risk,” “high risk,” or “unclear risk” (Li et al., 2023).

## Results

### Search results

**Figure 1:**
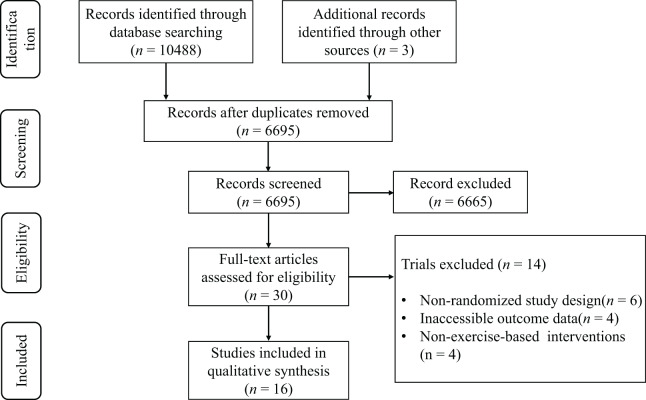
PRISMA flow diagram.

The preliminary database search revealed a total of 10,122 potentially pertinent articles. Following deduplication, 6,583 distinct records were preserved for evaluation. The review of titles and abstracts rejected 6,554 publications that failed to satisfy the inclusion criteria. A comprehensive evaluation of the remaining 29 articles led to the deletion of 14 studies for the following primary reasons: (1) nonrandomized study design (*n* = 6), (2) lack of outcome data (*n* = 4), and (3) treatments that were not independent campaigns (*n* = 4). In all, 15 articles (Bekris et al., 2022; Chatzinikolaou et al., 2010; Copeland, Consitt & Tremblay, 2002; Hackney, Premo & McMurray, 1995; Hough et al., 2011; Ispirlidis et al., 2008; Knatauskaitė et al., 2022; Kraemer et al., 2001; Lane & Hackney, 2015; McCaulley et al., 2009; Mirazizi, Rahmati-Ahmadabad & Fatolahi, 2022; Ronsen et al., 2001; Schumm et al., 2008; Shariat et al., 2014; Willoughby & Taylor, 2004) satisfied the inclusion criteria for the systematic review, as illustrated in the PRISMA flowchart (Fig. 1).

### Characteristics of included studies

Table 1 summarizes the main characteristics of the included studies. This study comprised 16 RCTs, encompassing a total sample size of 267 participants (189 males (70.8%) and 78 females (29.2%)). The sample exhibited a broad age range (15–69 years old, with 249 cases under 40 years old, accounting for 93.3%; 18 aged 40 and above, 6.7%). According to the participant classification framework (McKay et al., 2022), 146 subjects (54.7%) had a background in systematic training (including trained and highly trained levels), 112 (41.9%) were recreationally active, and a small number were classified as sedentary (9, 3.4%). The literature was published over a 30-year duration from 1995 to 2025. Geographically, North America was predominant, comprising six contributions from the United States (Hackney, Premo & McMurray, 1995; Kraemer et al., 2001; Lane & Hackney, 2015; McCaulley et al., 2009; Schumm et al., 2008; Willoughby & Taylor, 2004) and one from Canada (Copeland, Consitt & Tremblay, 2002). Europe followed with three from Greece (Bekris et al., 2022; Chatzinikolaou et al., 2010; Ispirlidis et al., 2008) and one each from the United Kingdom (Hough et al., 2011), Norway (Ronsen et al., 2001), and Germany (Knatauskaitė et al., 2022). Asia included one contribution each from Iran (Mirazizi, Rahmati-Ahmadabad & Fatolahi, 2022), Malaysia (Shariat et al., 2014) and China (Xu & Guo, 2025). Aerobic activity (Bekris et al., 2022; Copeland, Consitt & Tremblay, 2002; Hackney, Premo & McMurray, 1995; Hough et al., 2011; Ispirlidis et al., 2008; Knatauskaitė et al., 2022; Lane & Hackney, 2015; Mirazizi, Rahmati-Ahmadabad & Fatolahi, 2022; Ronsen et al., 2001) (9, 56.3%) and resistance training (Chatzinikolaou et al., 2010; Copeland, Consitt & Tremblay, 2002; Hough et al., 2011; Kraemer et al., 2001; McCaulley et al., 2009; Schumm et al., 2008; Shariat et al., 2014; Willoughby & Taylor, 2004; Xu & Guo, 2025) (9, 56.3%) constituted the primary intervention modalities in exercise intervention protocols, while two research (Copeland, Consitt & Tremblay, 2002; Hough et al., 2011) established multimodal exercise groups. The duration of exercise varied significantly (15–90 min/session), with training intensity distributed as follows: high-intensity interval training constituted 56.3% (9/16) (Bekris et al., 2022; Copeland, Consitt & Tremblay, 2002; Hackney, Premo & McMurray, 1995; Hough et al., 2011; Ispirlidis et al., 2008; Knatauskaitė et al., 2022; Lane & Hackney, 2015; McCaulley et al., 2009; Shariat et al., 2014), while moderate-intensity continuous training comprised 62.5% (10/16) (Chatzinikolaou et al., 2010; Copeland, Consitt & Tremblay, 2002; Knatauskaitė et al., 2022; Lane & Hackney, 2015; McCaulley et al., 2009; Mirazizi, Rahmati-Ahmadabad & Fatolahi, 2022; Ronsen et al., 2001; Schumm et al., 2008; Willoughby & Taylor, 2004; Xu & Guo, 2025). Notably, four studies (Copeland, Consitt & Tremblay, 2002; Knatauskaitė et al., 2022; Lane & Hackney, 2015; McCaulley et al., 2009) established a multi-intensity control experimental group.

Furthermore, among the trials encompassed in this study, 12 employed serum samples (Bekris et al., 2022; Chatzinikolaou et al., 2010; Copeland, Consitt & Tremblay, 2002; Hackney, Premo & McMurray, 1995; Ispirlidis et al., 2008; Lane & Hackney, 2015; McCaulley et al., 2009; Mirazizi, Rahmati-Ahmadabad & Fatolahi, 2022; Ronsen et al., 2001; Schumm et al., 2008; Xu & Guo, 2025), five utilized saliva samples (Hough et al., 2011; Knatauskaitė et al., 2022; Kraemer et al., 1998; Lane & Hackney, 2015; Shariat et al., 2014), and a mere one employed plasma samples (Hough et al., 2011). One trial utilized both serum and saliva samples (Lane & Hackney, 2015), while another employed both plasma and saliva samples (Hough et al., 2011). With regard to the measured indicators, 14 studies assessed total testosterone (Bekris et al., 2022; Copeland, Consitt & Tremblay, 2002; Hackney, Premo & McMurray, 1995; Hough et al., 2011; Knatauskaitė et al., 2022; Lane & Hackney, 2015; McCaulley et al., 2009; Mirazizi, Rahmati-Ahmadabad & Fatolahi, 2022; Ronsen et al., 2001; Schumm et al., 2008; Shariat et al., 2014; Willoughby & Taylor, 2004; Xu & Guo, 2025), while the remaining two measured free testosterone (Chatzinikolaou et al., 2010; Ispirlidis et al., 2008). With regard to the alterations in testosterone levels subsequent to exercise intervention, a total of ten trials documented a substantial increase in testosterone levels following exercise (Bekris et al., 2022; Chatzinikolaou et al., 2010; Copeland, Consitt & Tremblay, 2002; Hackney, Premo & McMurray, 1995; Hough et al., 2011; Lane & Hackney, 2015; McCaulley et al., 2009; Mirazizi, Rahmati-Ahmadabad & Fatolahi, 2022; Schumm et al., 2008; Willoughby & Taylor, 2004), five trials noted no statistically significant difference (Ispirlidis et al., 2008; Knatauskaitė et al., 2022; Kraemer et al., 2001; Ronsen et al., 2001; Xu & Guo, 2025), and a single trial reported a significant decrease in testosterone levels after exercise (Shariat et al., 2014).

### Risk of bias

**Figure 2:**
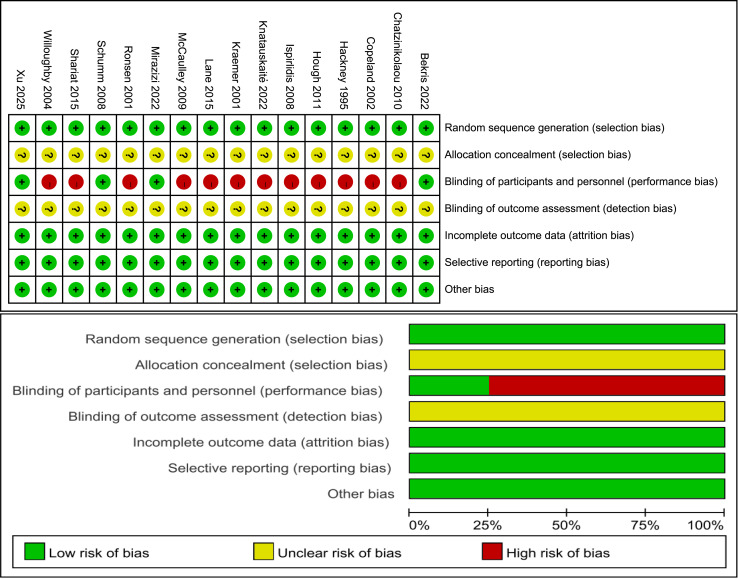
Risk of bias. Studies: Bekris et al. (2022), Chatzinikolaou et al. (2010), Copeland, Consitt & Tremblay (2002), Hackney, Premo & McMurray (1995), Hough et al. (2011), Ispirlidis et al. (2008), Knatauskaitė et al. (2022), Kraemer et al. (2001), Lane & Hackney (2015), McCaulley et al. (2009), Mirazizi, Rahmati-Ahmadabad & Fatolahi (2022), Ronsen et al. (2001), Schumm et al. (2008), Shariat et al. (2014), Willoughby & Taylor (2004), Xu & Guo (2025).

Figure 2 illustrates the classification of the included studies into low, moderate, or high quality based on the following criteria: (1) a trial was considered to be of low quality if the risk of bias for randomization or allocation concealment was high, while the risk for other items was negligible; (2) a trial was considered to be of high quality if the risk of bias for randomization and allocation concealment was low while the risk of bias for all other items was low or unclear; and (3) a trial was considered to be of moderate quality if the trial did not meet the criteria for high or low risk (Li et al., 2023). Specifically, three trials had a low risk of bias (Bekris et al., 2022; Mirazizi, Rahmati-Ahmadabad & Fatolahi, 2022; Schumm et al., 2008) and 12 trials had a moderate risk (Chatzinikolaou et al., 2010; Copeland, Consitt & Tremblay, 2002; Hackney, Premo & McMurray, 1995; Hough et al., 2011; Ispirlidis et al., 2008; Knatauskaitė et al., 2022; Kraemer et al., 2001; Lane & Hackney, 2015; McCaulley et al., 2009; Ronsen et al., 2001; Shariat et al., 2014; Willoughby & Taylor, 2004).

## Discussion

Testosterone has long been a focal point of study regarding exercise and acute endocrine responses, particularly due to its role as a principal anabolic hormone secreted in reaction to physical activity, alongside the enhanced production of numerous catabolic and anabolic hormones. Variations in testosterone levels have garnered significant focus in research concerning male health and reproductive function, particularly in relation to acute aerobic and resistance training. The post-exercise recovery phase is a significant focus of investigation in exercise physiology, especially regarding the restoration of testosterone levels. Testosterone, the principal male sex hormone, participates in numerous physiological processes, including muscle development and recuperation. Post-exercise, particularly during high-intensity training, there is typically a temporary increase in testosterone levels, succeeded by a gradual reversion to baseline levels (Zitzmann & Nieschlag, 2001). The recuperation process may be affected by several aspects, including exercise intensity, exercise kind, duration, the individual’s physiological status, and the source (saliva/blood) and baseline amount of testosterone.

This study systematically analyzed 15 RCTs to examine the dynamic effects of acute exercise on testosterone levels. Certain studies noted a temporary increase in testosterone following exercise, while others indicated a sustained testosterone level below baseline during the recovery phase. This discrepancy may arise from the interplay of exercise intervention factors, participant characteristics, and methodological variations.

### Effect of exercise type on acute post-exercise testosterone levels

Various exercise type exhibit considerable variability in the modulation of testosterone levels, which may arise from the interplay between activity-specific physiological stress and endocrine feedback systems. Aerobic exercise influences the dynamic equilibrium of testosterone secretion *via* multifaceted mechanisms, while acute exercise can directly enhance the steroidogenic potential of testicular mesenchymal stromal cells through augmented blood supply (Moir et al., 2024) and sympathetic stimulation (Watt & Spriet, 2004). Testosterone recovery post-aerobic exercise is notably swift, typically reverting to baseline levels within 1 h (Hackney, Premo & McMurray, 1995), likely due to its comparatively low metabolic strain, prompt reestablishment of energy substrates, and the ephemeral nature of oxidative stress (Ammar et al., 2020).

The neuroendocrine modulation induced by resistance training *via* mechanical loading stimuli is distinctive. Microdamage in muscle fibers caused by external loads activates protein synthesis pathways (Grgic et al., 2020) and directly promotes testosterone release while enhancing myocyte sensitivity to anabolic signals (Griggs et al., 1986). While acute resistance exercise markedly elevates testosterone concentrations, its recovery kinetics exhibit prolonged fluctuation cycles, with certain studies noting recovery duration extending to hours or beyond from baseline levels (Tremblay, Copeland & Van Helder, 2004), potentially linked to modified myocyte membrane permeability induced by resistance training (Wernbom et al., 2012), enhanced glycogen depletion (Hokken et al., 2021), and an augmented response of the HPA axis (Athanasiou, Bogdanis & Mastorakos, 2023). The duration of HPA axis activation is extended. The variation in hormonal response unique to exercise types indicates that the immediate effects of different exercise type on the HPG axis, along with the outcomes of chronic adaptation, must be integrated when formulating training regimens.

### Effect of exercise intensity on acute post-exercise testosterone levels

Overall, circulating total and free testosterone levels rise immediately post-exercise and revert to or fall below baseline within 30 min (Kraemer et al., 2006; Ratamess et al., 2005); however, these fluctuations are significantly affected by the selected exercise intensity. A relative intensity or total work output is necessary to elicit a testosterone response. The intensity of exercise influences the temporal dynamics of testosterone metabolism by variably regulating the neuroendocrine system. Moderate to high-intensity exercise can elevate peak blood testosterone at the conclusion of exercise by stimulating the HPG axis (Kumagai et al., 2018; Lane & Hackney, 2015), whereas excessive intensity may lead to a substantial decrease in recovery testosterone levels due to over-activation of the HPA axis (Hackney, Premo & McMurray, 1995; Shariat et al., 2014). When duration or repetitions remain constant, higher intensity exercise provokes a more substantial testosterone response. Raastad, Bjøro & Hallén (2000) exhibited a more pronounced acute response in testosterone during a high-intensity regimen relative to a moderate-intensity regimen. Meta-analytic evidence indicates that moderate- and high-intensity exercise significantly elevate testosterone levels in men (D’Andrea et al., 2020), while low-intensity exercise necessitates an extended duration to induce a notable increase in testosterone (Galbo et al., 1977), highlighting the crucial modulation of hormonal response by the interaction of intensity and duration.

When exercise intensity surpasses physiological thresholds, it induces an imbalance in endocrine homeostasis. Although male endurance athletes experience acute increases in testosterone and cortisol immediately following fatigue-inducing running, free testosterone remains below baseline levels during the subsequent 24-h recovery period (Daly et al., 2005). In strength training, total and free testosterone levels in men diminished during the initial two days following high-intensity deep squats (10 sets of 10 repetitions at 70% of one repetition maximum (1 RM) or 20 sets of one repetition at 100% of 1 RM) (Häkkinen & Pakarinen, 1993). A comparable pattern has been confirmed in international rugby players during post-competition surveillance, indicating that total testosterone levels required 36 h to revert to pre-competition values (Cunniffe et al., 2010). This may result from high-intensity exercise activating the HPA axis, which elevates cortisol levels and creates an antagonistic connection with testosterone. This hormonal imbalance may result in suboptimal testosterone levels by suppressing testosterone production (Choi, Chung & Lee, 2012). Consequently, accurate regulation of exercise intensity thresholds is crucial for sustaining hormonal equilibrium.

### Effect of sample origin on acute post-exercise testosterone levels

In clinical practice, the identification of testosterone levels using biomarkers relies on two matrices, blood and saliva, which exhibit notable methodological discrepancies. In the study by Hough et al. (2011), Lane & Hackney (2015), simultaneous testing of serum and saliva samples revealed that acute exercise induces significant changes in serum and salivary testosterone levels. This change demonstrates a consistent pattern of elevation during both moderate-intensity and high-intensity exercise. Notably, under high-intensity exercise conditions, the response of salivary testosterone levels shows a stronger correlation with serum testosterone levels. Serum testing, the conventional gold standard (Fink, Bentzen & Horie, 2025), possesses a robust capacity to eliminate interference from other steroid hormones (Viette et al., 2011). The clinical application of this method is constrained by several factors: it necessitates the participation of healthcare professionals, and there are obstacles to the practicality of sampling for dynamic monitoring during exercise. Additionally, samples must be transported at -80°C and analyzed within 24 h, which presents a challenge for accessibility in resource-limited regions (Patjamontri et al., 2023).

In contrast, saliva testing has shown distinct benefits as a non-invasive option for youngsters, elderly individuals, and large-scale epidemiological screening (Horie, 2010; Xia et al., 2014). However, the precision and dependability of salivary tests are frequently influenced by many circumstances. Research indicates that while saliva testosterone is effective for detecting large doses of exogenous testosterone (Schönfelder et al., 2011), it poses challenges for accurately measuring endogenous testosterone levels. In twelve trained male subjects, low-intensity exercise resulted solely in an increase in serum total testosterone without notable alterations in salivary indices, while both exhibited a coordinated response during moderate- and high-intensity exercise (Lane & Hackney, 2015), indicating that salivary testing possesses greater ecological validity for acute hormonal fluctuations elicited by high-intensity exercise.

### Effect of gender on acute post-exercise testosterone levels

Gender disparities markedly influence the kinetic profile of testosterone caused by exercise stress. Signals for gonadal testosterone synthesis and secretion originate in the hypothalamus, where specialized neurons synthesize and release gonadotropin-releasing hormone (GnRH). This hormone enters the anterior pituitary gland, stimulating the production of gonadotropins and the release of luteinizing hormone, which in turn promotes testosterone synthesis in testicular mesenchymal cells in males and in ovarian membrane cells in females (Kim, 2007). In females, testosterone is converted into oestradiol in granulosa cells neighbouring the membrane cells. Evidence indicates that a single session of high-intensity exercise causes immediate increases in serum total and free testosterone levels in male individuals, with peaks generally observed right after exercise and reverting to baseline or lower levels within 30 min (Copeland, Consitt & Tremblay, 2002; Kraemer et al., 1998; Lane & Hackney, 2015; Ronsen et al., 2001; Schumm et al., 2008; Willoughby & Taylor, 2004). It is notable that the magnitude of the hormonal response was influenced by the exercise regimen. The molecular mechanisms driving the exercise-induced testosterone response in women differ fundamentally from those in men. The primary site of testosterone production is the testicular interstitial cells, located solely in the testis. In women, androgen fluctuations may pertain to the dehydroepiandrosterone (DHEA) conversion pathway secreted by the reticular zona of the adrenal cortex, which largely accounts for the circulating testosterone levels in men, approximately ten times greater than those in women (Kvorning et al., 2007).

Limited research has directly investigated the impact of specific acute exercise on the post-exercise testosterone response in women. While some studies have documented significant increases in free or total testosterone following resistance training (Copeland, Consitt & Tremblay, 2002; Nindl et al., 2001), there remains considerable variability in findings across similar studies, with most reporting no acute elevation in post-exercise testosterone levels (Kraemer et al., 1993; Linnamo et al., 2005). Linnamo et al. (2005) established that under three distinct loading conditions: maximal (10 RM), submaximal (70% of 10 RM), and explosive (40% of 10 RM), there was no alteration in testosterone following five sets of 10 repetitions of sit-ups, bench press, and leg press. In males, identical maximal loading conditions led to a substantial elevation in testosterone (Linnamo et al., 2005). Kraemer et al. (1993) similarly shown that altering rest time, length, or load while maintaining a constant total volume during high-intensity exercise did not yield elevated post-exercise testosterone concentrations compared to baseline in women. In males, GnRH analogues suppress circulating luteinizing hormone, subsequently inhibiting testicular interstitial cell activity. This results in depot testosterone levels and obstructs acute resistance exercise-induced elevations in total and free testosterone. Consequently, testicular interstitial cells seem to be pivotal for the significant surge in post-exercise testosterone, potentially explaining the lack of testosterone responsiveness to resistance exercise in females (Vingren et al., 2010). Nonetheless, there is an insufficiency of robust, research-consistent evidence to substantiate this concept, and systematic validation *via* standardized experimental paradigms is urgently required.

### Effect of age on acute post-exercise testosterone levels

The immediate effects of exercise on testosterone levels are significantly affected by age. According to Knatauskaitė’s research, acute exercise does not induce substantial alterations in testosterone levels among adolescent children, irrespective of gender (boys or girls) or the intensity of the exercise. Even post-puberty, when baseline testosterone levels begin to rise, no exercise-induced increase in testosterone is observed (Pullinen et al., 2002, 1998). Pullinen et al. (1998) established that following vigorous squatting exercises, plasma norepinephrine levels in teenage boys, along with total testosterone levels, were lower than those in adult males, and no association between these two responses was detected. testosterone levels in high school-aged subjects did not exhibit significant changes following the same workout regimen as that of college-aged subjects (Fahey et al., 1976). The restricted post-exercise testosterone response in adolescent children may result from the testes’ inability to swiftly augment testosterone secretion, insufficient metabolic stimulation during exercise, and the observation that adolescent athletes with extensive weightlifting training experience exhibit a more pronounced testosterone response than novice athletes during exercise (Kraemer et al., 1992).

The extent of exercise-induced alterations in testosterone levels appears to diminish with age following sexual maturity, particularly evident for free testosterone. The disparity between free and total testosterone levels may be attributed to elevated sex hormone-binding globulin (SHBG) concentrations and diminished albumin concentrations associated with ageing (Rodriguez et al., 2007). Acute post-exercise testosterone responses seem to be less significant in middle-aged and older adults (≥38 years) compared to younger adults (20–36 years) (Baker et al., 2006). Kraemer et al. (1998), when comparing acute post-exercise hormonal reactions in younger and older men, discovered that there were age-related changes in endocrine responses to exercise, with the most pronounced response being that of free testosterone. Furthermore, for young men, evening exercise may have a more pronounced effect on hormones compared to morning exercise (Xu & Guo, 2025). This indicates that the body can elicit physiological stimulation of the endocrine system *via* acute exercise, resulting in variations in testosterone levels, which are affected by age, as younger males exhibit a greater capacity for exercise-induced testosterone responses compared to older males. Additionally, the timing of physical activity can also have a significant impact on the body’s response.

### Limitation

This study has several limitations. The included studies exhibited significant heterogeneity in exercise intervention protocols and participant characteristics, and testosterone measurement methods and metrics varied, collectively limiting the direct comparability of results. As only one study comparing exercise at different time points was identified, it was not discussed separately. In light of the methodological heterogeneity that may compromise the interpretability of quantitative meta-analysis results, a meta-analysis was not conducted. Instead, a qualitative synthesis approach was employed to systematically present the evidence landscape. Future research endeavors must entail the execution of larger, rigorously designed studies that employ standardized measurement criteria to validate the acute effects of specific exercise parameters on testosterone levels.

## Conclusion

This study systematically reviewed RCT evidence on testosterone kinetics after acute exercise and revealed the complexity of changes in testosterone levels after exercise interventions: heterogeneity was mainly attributed to exercise mode, exercise intensity, sample source, and subject characteristics (sex and age). The findings indicate that variations in post-exercise testosterone levels may serve as a biological marker for evaluating training adaptation and recovery status; however, individual factors such as gender and age, along with methodological heterogeneity issues like sample source control, must be taken into account in the formulation of exercise programs. This establishes a theoretical foundation for precise exercise prescription, which is beneficial for enhancing training oversight and chronic illness management approaches. Future research must standardize experimental design to mitigate the impact of confounding variables.

## Supplemental Information

10.7717/peerj.20615/supp-1 Supplemental Information 1 PRISMA checklist.

## Additional Information and Declarations

### Data Availability

The following information was supplied regarding data availability:

The raw data are available in Table 1 and Figs. 1 and 2.
